# Genetic analysis of egg production traits in turkeys (*Meleagris gallopavo*) using a single-step genomic random regression model

**DOI:** 10.1186/s12711-021-00655-w

**Published:** 2021-07-20

**Authors:** Hakimeh Emamgholi Begli, Lawrence R. Schaeffer, Emhimad Abdalla, Emmanuel A. Lozada-Soto, Alexandra Harlander-Matauschek, Benjamin J Wood, Christine F. Baes

**Affiliations:** 1 Centre for Genetic Improvement of Livestock, Department of Animal Biosciences, University of Guelph, Guelph, N1G 2W1 Canada; 2 Hybrid Turkeys, A Hendrix Genetics Company, Kitchener, N2K 3S2 Canada; 3 School of Veterinary Science, University of Queensland, Gatton Campus, Brisbane, QLD Australia; 4 Campbell Centre for the Study of Animal Welfare, Department of Animal Biosciences, University of Guelph, Guelph, N1G 2W1 Canada; 5 Institute of Genetics, Vetsuisse Faculty, University of Bern, 3001 Bern, Switzerland

## Abstract

**Background:**

Egg production traits are economically important in poultry breeding programs. Previous studies have shown that incorporating genomic data can increase the accuracy of genetic prediction of egg production. Our objective was to estimate the genetic and phenotypic parameters of such traits and compare the prediction accuracy of pedigree-based random regression best linear unbiased prediction (RR-PBLUP) and genomic single-step random regression BLUP (RR-ssGBLUP). Egg production was recorded on 7422 birds during 24 consecutive weeks from first egg laid. Hatch-week of birth by week of lay and week of lay by age at first egg were fitted as fixed effects and body weight as a covariate, while additive genetic and permanent environment effects were fitted as random effects, along with heterogeneous residual variances over 24 weeks of egg production. Predictions accuracies were compared based on two statistics: (1) the correlation between estimated breeding values and phenotypes divided by the square root of the trait heritability, and (2) the ratio of the variance of BLUP predictions of individual Mendelian sampling effects divided by one half of the estimate of the additive genetic variance.

**Results:**

Heritability estimates along the production trajectory obtained with RR-PBLUP ranged from 0.09 to 0.22, with higher estimates for intermediate weeks. Estimates of phenotypic correlations between weekly egg production were lower than the corresponding genetic correlation estimates. Our results indicate that genetic correlations decreased over the laying period, with the highest estimate being between traits in later weeks and the lowest between early weeks and later ages. Prediction accuracies based on the correlation-based statistic ranged from 0.11 to 0.44 for RR-PBLUP and from 0.22 to 0.57 for RR-ssGBLUP using the correlation-based statistic. The ratios of the variances of BLUP predictions of Mendelian sampling effects and one half of the additive genetic variance ranged from 0.17 to 0.26 for RR-PBLUP and from 0.17 to 0.34 for RR-ssGBLUP. Although the improvement in accuracies from RR-ssGBLUP over those from RR-PBLUP was not uniform over time for either statistic, accuracies obtained with RR-ssGBLUP were generally equal to or higher than those with RR-PBLUP.

**Conclusions:**

Our findings show the potential advantage of incorporating genomic data in genetic evaluation of egg production traits using random regression models, which can contribute to the genetic improvement of egg production in turkey populations.

## Background

Egg production traits are an important component of selection indexes in commercial turkey flocks. Since the heritability of egg production traits changes throughout the laying period [1], a single value of cumulated egg production over the entire laying period does not sufficiently describe it. The efficiency of genetic selection for egg production traits can be improved by using longitudinal models, which enable a more correct consideration of genetic variances and covariances over time. Random regression (RR) models have been used successfully for genetic evaluation of longitudinal traits in various livestock species [2–4]. Such models have proven to be useful for the genetic improvement of egg production and persistency of egg laying hens [1]. The advantage of the RR model is its ability to predict breeding values for cumulative egg production throughout the laying period and at any specific day of production [5] and to evaluate persistency of production.

Incorporating genomic data into breeding programs has improved selection accuracy in several species [6–8]. Single- and multi-step approaches have been used for genomic evaluation but single-step methods are preferred because they use all available genotypic and phenotypic information. The single‐step genomic best linear unbiased prediction (ssGBLUP) method integrates the genomic relationship matrix with the pedigree relationship matrix, thus allowing inclusion of all genotyped and non-genotyped animals. This simultaneous use of relationships enables unbiased prediction of breeding values [9, 10]. In addition to the advantages of the single-step method, its straightforward extension to more complex models, such as RR models for longitudinal traits, makes it particularly useful for genomic evaluation [11, 12].

The use of ssGBLUP based on RR has been shown to increase the accuracy of genomic predictions for longitudinal traits in dairy cows [12, 13]. In order to maximize genetic gain and accuracy of the prediction of breeding values in turkeys, the implementation of genomic approaches over traditional methods by using RR requires investigation. The goal of our study was to estimate variance components of egg production traits in turkeys over time and to compare the prediction accuracy of pedigree-based random regression BLUP (RR-PBLUP) and genomic single-step random regression BLUP (RR-ssGBLUP).

## Methods

### Data

Egg production was recorded on 7422 hens from a female line over a 9-year period. The hens laid in individual trap nests and egg numbers were recorded on a daily basis. The trait analyzed was the cumulative number of hatchable eggs laid in trap nests per week over 24 weeks from the date of the first egg for each bird. Thus, hens started laying at different ages, but all hens completed egg laying in 24 weeks. This is similar to milk yield in dairy cattle, which begins at calving and continues until the cow produces less than a certain amount of milk, at which point it is dried off, or until it reaches a certain lactation length, e.g. 305 days. Cows milk for different numbers of days from 240 to 400 days, and begin to milk at different ages at calving, but the RR model accommodates these differences, as well as a variable number of test measures per cow. Similarly, in hens, production starts with the first egg and ends with the last egg (in a specified week), for hens of different ages at first egg, and the RR model can accommodate this. Hens will typically take one to two weeks to begin laying eggs after exposure to longer light periods is initiated (light date) to stimulate the on-set of lay. The production curves of hens are more similar when production is described relative to the first day of lay. Hens that start lay a week or two after the light date are not desired, but that is a different trait than egg production and will be called delay time (DT). Selection against hens with a long DT regardless of their estimated breeding values for egg production is beyond the scope of this paper. Normal practice for commercial producers is to look at eggs produced within 26 weeks from the lighting date regardless of when a hen begins laying, which is adequate when total egg production is the target, but not if egg production curves are needed to identify different shapes of production, as in this study.

### Statistical analyses

A phenotypic variance–covariance matrix of order 24 was estimated using the egg production data recorded by week using the R software [14]. Covariance functions were fitted to this matrix and reduced orders of fit were examined. A third order polynomial was found to fit the 24-by-24 matrix sufficiently well. Hence, third order Legendre polynomials (i.e. 4 covariates) were used in the RR models for the genetic and permanent environmental components.

In total, 3561 animals in this population were genotyped using the Illumina 65 K single nucleotide polymorphism (SNP) panel. Genotyping quality control consisted of filtering out SNPs that had a minor allele frequency lower than 5%, a call rate lower than 95%, or that were localised in non-autosomal regions. In total, 3178 birds and 47,387 SNPs passed quality control and were used for the analysis.

The data were analyzed with the following RR model:

$$\begin{aligned}{\mathbf{y}}_{\mathbf{i}\mathbf{j}\mathbf{m}\mathbf{t}}&={\left(H\times W\right)}_{it}+(W\times {AFE)}_{mt} +{{b}_{t}\left(Bwt\right)}_{t} \\ & \quad +\sum_{k=0}^{3}{\alpha }_{kj}{{\varnothing }}_{k}\left(t\right)+\sum_{k=0}^{3}{p}_{kj}{{\varnothing }}_{k}\left(t\right)+{e}_{ijmt},\end{aligned}$$

where ${\mathbf{y}}_{\mathbf{i}\mathbf{j}\mathbf{m}\mathbf{t}}$ is the vector of observations for weekly egg production; $H$ is the hatch-week of birth, which describes the hens born at the same time and raised under the same conditions (i.e. contemporaries); week of lay $W$ ($t$ = 1 to 24) and age at first egg ($AFE$), which was categorized into three groups (i.e. 29 to 31, 32 to 34, and > 34 weeks) are fixed effects; body weight at start of lay ($Bwt$) is a covariate with a different slope for each week of lay; for each hen $j$, ${\alpha }_{kj}$ and ${p}_{kj}$ are the $k$^th^ random regression coefficients for additive genetic and permanent environmental effects, respectively; ${{\varnothing }}_{k}\left(t\right)$ is the $k$^th^ Legendre polynomial covariate for the observation of individual $j$ in week $t$; third order Legendre polynomials of weeks of production were fitted for both additive genetic and permanent environmental effects; ${e}_{ijmt}$ is the residual variance, which was assumed to be heterogeneous and divided into four periods (1 to 4, 5 to 13, 14 to 19, and 20 to 24 weeks).

The matrix representation of the model is:

$$\mathbf{y}=\mathbf{X}\mathbf{b}+\mathbf{Q}\mathbf{a}+\mathbf{Z}\mathbf{p}+\mathbf{e},$$

where $\mathbf{y}$ is the vector of observations; $\mathbf{X}$, $\mathbf{Q}$ and $\mathbf{Z}$ are incidence matrices corresponding to the fixed effects ($\mathbf{b}$), additive genetic effects ($\mathbf{a}$), and permanent environmental effects ($\mathbf{p}$); $\mathbf{e}$ is the vector of residuals. For the pedigree-based model, denoted RR-PBLUP, it was assumed that:

$$Var\left[ {\begin{array}{*{20}c} {\mathbf{a}} \\ {\mathbf{p}} \\ {\mathbf{e}} \\ \end{array} } \right] = \left[ {\begin{array}{*{20}c} {{\mathbf{A}} \otimes {\mathbf{C}}} & 0 & 0 \\ 0 & {{\mathbf{I}} \otimes {\mathbf{P}}} & 0 \\ 0 & 0 & {\mathbf{R}} \\ \end{array} } \right],$$

where $\mathbf{I}$ is an identity matrix with dimensions equal to the number of hens, $\otimes$ is the Kronecker product, $\mathbf{C}$ and $\mathbf{P}$ are (co)variance matrices of additive genetic and permanent environmental regression coefficients, respectively. The size of $\mathbf{C}$ and $\mathbf{P}$ is (4 × 4), $\mathbf{R}$ is a diagonal matrix of four residual variances corresponding to the four lay periods, of order equal to the number of observations, and $\mathbf{A}$ is the numerator relationship matrix based on pedigree information of order equal to the number of animals in the pedigree file.

The variance and covariance components for the random effects for weekly egg number were obtained as $${\mathbf{\upphi}}^{\prime}{\mathbf{C}}{\mathbf{\upphi}}$$and $${\mathbf{\upphi}}^{\prime}{\mathbf{P}}{\mathbf{\upphi}}$$, where $${\varvec{\upphi}}
$$ is the matrix of Legendre polynomial covariates per order (4 by 24). The resulting covariance matrices are of order 24-by-24.

For RR-ssGBLUP, the inverse of the numerator relationship matrix ($\mathbf{A}$) in the traditional mixed model equations (MME) was replaced by the ${\mathbf{H}}^{-1}$ matrix, which is defined as follows [15]:

$${\mathbf{H}}^{-1}={\mathbf{A}}^{-1}+\left[\begin{array}{cc}0& \quad 0\\ 0& \quad {\mathbf{G}}^{-1}-{\mathbf{A}}_{22}^{-1}\end{array}\right],$$

where ${\mathbf{A}}^{-1}$ is the inverse numerator relationship matrix for all animals, ${\mathbf{A}}_{22}^{-1}$ is the inverse of a pedigree-based relationship matrix for genotyped animals only, and ${\mathbf{G}}^{-1}$ is the inverse genomic relationship matrix. $\mathbf{G}$ was calculated by using the first method proposed by VanRaden [16], with ${\mathbf{G}}_{k}$ blended as $0.95\mathbf{G}+0.05{\mathbf{A}}_{22}$ to obtain a non-singular matrix.

Analyses for the estimation of genetic parameters were performed using a Bayesian approach via Gibbs sampling implemented in the GIBBS1F90 module of the BLUPF90 software [17]. The Gibbs sampler was run for 200,000 rounds, with the first 20,000 considered as burn-in and then every 50th sample saved for posterior analysis. Posterior means and standard deviations were calculated to obtain estimates of variance components. Convergence of the posterior parameters was assessed by visual inspection of trace plots of posterior distributions generated by the Coda R package [18]. Only the estimates of the variances and covariances obtained with RR-ssGBLUP are presented.

### Accuracy of prediction of breeding values

Two statistics were calculated to assess the improvement in accuracy from incorporating genomic information. The first statistic was the correlation between estimated breeding values and phenotypic data. For this purpose, the data were divided into reference and validation subsets. Approximately 10% of the youngest turkeys were assigned to the validation group, for which predictions were made, while the remaining 90% were used to train the model. Prediction accuracy of estimated breeding values in the validation data was estimated as the Pearson correlation coefficient between estimated pedigree breeding values (EBV) or genomic breeding values (GEBV) and phenotypes corrected for fixed effects divided by the square root of heritability obtained with RR-ssGBLUP [19].

The second statistic, not previously published in the literature, was developed as follows. The use of genomic data in genetic evaluation aims at improving the accuracy of the Mendelian sampling effect, so that full-sibs may be ranked. Thus, Mendelian sampling effects were predicted by BLUP as:

$$\widehat{ms}_{i} = EBV_{i} - 0.5\left( {EBV_{sire} + { }EBV_{dam} } \right)$$

where ${EBV}_{i}$, ${EBV}_{sire}$, and ${EBV}_{dam}$ are the BLUP estimated breeding values of individual $i$ and of its sire and dam, respectively, invoking the invariance property of BLUP. BLUP theory says that the predicted variance of the Mendelian sampling effects approaches the expected variance as the estimates become more accurate (or as the prediction error variance decreases) [20]. Thus, the ratio of the variance of the estimated Mendelian sampling effects to its expected variance, which is one half of the additive genetic variance, gives a measure of the general accuracy of the Mendelian sampling estimates, as follows:

$$\upnu =\text{Var}(\mathbf{m}\mathbf{s})/(0.5\text{Var}(\mathbf{a})).$$

As the predicted Mendelian sampling effects become more accurate, the ratio should become larger and $\upnu$ should range from 0 to 1. Only animals with records and both parents known were used. Data do not need to be partitioned into reference and validation subsets, and the statistic does not directly involve phenotypic records, $\mathbf{y}$.

## Results and discussion

Means, standard deviations, and coefficients of variation (CV) of weekly egg numbers over 24 weeks are in Table 1. The smallest mean number of eggs (3.52) and the highest CV were obtained in the last week, while the lowest CV was obtained in the first week. Previous studies that were conducted in layers reported greater variation during the early and final production periods [21, 22]. These differences between studies may be due to our definition of weekly egg production and to the difference in the production curves of layers compared to turkeys. Here, weekly egg production was defined from age at first egg, whereas the previous study on layers by Anang et al. [21] used the lighting date, but not all hens start laying on the lighting date. Analysis of egg production curves provides information regarding the peak, decline, and persistency of lay, which helps to select birds at young ages, effectively manage the farm, and increase egg production [23].

**Table 1:** Descriptive statistics for the egg production traits

Week	Number of hens	Average egg production	Standard deviation	Coefficient of variation (%)
1	7422	5.21	1.34	25.7
2	7351	5.26	1.47	28.0
3	7332	5.30	1.48	27.9
4	7310	5.22	1.49	28.6
5	7296	5.16	1.51	29.3
6	7284	5.06	1.53	30.2
7	7259	4.94	1.53	30.9
8	7231	4.85	1.54	31.7
9	7205	4.77	1.52	31.9
10	7184	4.71	1.51	32.1
11	7132	4.58	1.50	32.6
12	7055	4.50	1.49	33.0
13	7022	4.38	1.47	33.6
14	6972	4.29	1.47	34.1
15	6937	4.16	1.45	34.8
16	6883	4.09	1.44	35.1
17	6823	3.98	1.44	36.1
18	6683	3.91	1.43	36.5
19	6592	3.88	1.41	36.5
20	6427	3.84	1.40	36.3
21	6081	3.82	1.37	35.9
22	5719	3.82	1.35	35.4
23	5101	3.73	1.30	34.9
24	3478	3.53	1.30	36.9

The egg production curve was divided into 24 weeks of 7 days, starting with the day of first egg

### Genetic parameters

Table 2 presents posterior means and highest 95% posterior density (HPD) intervals for the additive genetic and permanent environmental variances for the weekly egg production traits. All parameters were estimated with precision, as reflected by the narrow 95% HPD intervals. Estimates indicate that environmental differences in the studied weeks have a large influence on egg production. Estimates of covariance matrices, $\mathbf{C}$ and $\mathbf{P}$, for additive genetic and permanent environmental effects are in Tables 3 and 4 along with the heterogeneous residual variances in Table 5.

**Table 2:** Posterior means and 95% highest probability density (HPD) intervals for additive genetic and permanent environmental ($\mathbf{P}\mathbf{E}$) variances and of the heritability for egg number for over 24 weeks of lay

Week	Genetic variance		PE variance		Heritability	
	Estimate	HPD	Estimate	HPD	Estimate	HPD
1	0.19	0.15–0.22	0.55	0.52–0.58	0.09	0.08–0.10
2	0.19	0.16–0.23	0.47	0.45–0.5	0.10	0.08–0.11
3	0.21	0.17–0.25	0.48	0.45–0.5	0.10	0.09–0.12
4	0.23	0.19–0.27	0.52	0.49–0.55	0.11	0.10–0.13
5	0.26	0.22–0.3	0.57	0.54–0.6	0.12	0.10–0.13
6	0.28	0.24–0.33	0.61	0.58–0.64	0.12	0.11–0.14
7	0.31	0.26–0.35	0.63	0.6–0.66	0.13	0.12–0.15
8	0.33	0.29–0.37	0.64	0.61–0.67	0.14	0.13–0.15
9	0.35	0.31–0.39	0.63	0.61–0.66	0.15	0.14–0.16
10	0.37	0.33–0.41	0.61	0.59–0.64	0.16	0.15–0.17
11	0.39	0.35–0.42	0.59	0.57–0.62	0.16	0.15–0.17
12	0.40	0.37–0.44	0.57	0.55–0.59	0.17	0.16–0.18
13	0.42	0.39–0.45	0.55	0.53–0.57	0.19	0.18–0.20
14	0.43	0.4–0.47	0.54	0.52–0.56	0.20	0.19–0.21
15	0.44	0.41–0.48	0.53	0.51–0.55	0.20	0.19–0.21
16	0.45	0.41–0.49	0.52	0.5–0.55	0.21	0.20–0.22
17	0.45	0.41–0.49	0.51	0.49–0.54	0.21	0.20–0.22
18	0.45	0.39–0.5	0.50	0.47–0.53	0.22	0.20–0.23
19	0.43	0.36–0.49	0.48	0.44–0.52	0.21	0.19–0.23
20	0.40	0.32–0.48	0.45	0.39–0.51	0.21	0.18–0.23
21	0.37	0.25–0.47	0.42	0.33–0.51	0.19	0.15–0.23
22	0.32	0.16–0.47	0.40	0.27–0.53	0.18	0.11–0.22
23	0.27	0.05–0.48	0.41	0.22–0.61	0.15	0.04–0.22
24	0.21	0.00–0.51	0.50	0.21–0.81	0.12	0.00–0.21

**Table 3:** Estimates of covariance matrices for order three Legendre polynomials for the additive genetic effects

$$\left[\begin{array}{cccc}0.62& 0.06& -0.08& -0.01\\ 0.06& 0.04& -0.02& -0.02\\ -0.08& -0.02& 0.02& 0.00\\ -0.01& -0.02& 0.00& 0.01\end{array}\right]$$

**Table 4:** Estimates of covariance matrices for order three Legendre polynomials for the permanent environmental effects

$$\left[\begin{array}{cccc}0.73& -0.03& -0.12& 0.04\\ -0.03& 0.18& -0.01& -0.06\\ -0.12& -0.01& 0.09& -0.01\\ 0.04& -0.06& -0.01& 0.05\end{array}\right]$$

**Table 5:** Estimates of residual variances

Period	Residual variances
Weeks 1 to 4	1.33
Weeks 5 to 12	1.38
Weeks 13 to 17	1.21
Weeks 18 to 24	1.10

Estimates of heritabilities and genetic correlations for egg production by using pedigree information for the selected weeks are in Table 6. Previously reported heritability estimates for monthly egg production using random regression models in turkeys ranged from 0.08 to 0.12 [24]. In our study, estimates of heritability for weekly egg production were lower, ranging from 0.09 to 0.22, which is consistent with estimates obtained with spline models in laying hens [25]. For the first four weeks, our estimates of heritability for egg production were lower than those reported for White Leghorn hens and native chickens for the early weeks of production [22]. For early egg production, Biscarini et al. [26] found heritabilities of 0.36 for total number of eggs produced from 17 to 24 weeks of age in laying hens; Nurgiartiningsih et al. [27] reported heritabilities of 0.32 and 0.38 for number of eggs produced in the first month of lay in two lines of White Leghorn hens. The high heritability estimate for early production was strongly influenced by variation in the rate of lay before the production peak as well as variation in age at sexual maturity [27]. The lower estimates of heritability for the early weeks in our study could be partially explained by differences in the models used for the analysis and in the definition of egg production traits. In our study, $AFE$ was considered as a fixed effect in the model, and egg production was defined from age at first egg. Estimates of heritability were moderately high for weeks 18 to 20 (h^2^ = 0.22 ± 0.02) of production and decreased during the last four weeks (h^2^ = 0.12 ± 0.01).

**Table 6:** Estimates ± standard error of heritability (on the diagonal in italics) and of genetic (above diagonal) and phenotypic correlations (below diagonal) for egg number in selected weeks of egg production

	Week 1	Week 4	Week 8	Week 12	Week 16	Week 20	Week 24
Week 1	*0.09* ± *0.01*	0.88 ± 0.04	0.75 ± 0.06	0.70 ± 0.06	0.67 ± 0.06	0.67 ± 0.06	0.76 ± 0.05
Week 4	0.27 ± 0.01	*0.11* ± *0.01*	0.96 ± 0.01	0.88 ± 0.02	0.78 ± 0.04	0.74 ± 0.04	0.87 ± 0.02
Week 8	0.18 ± 0.01	0.35 ± 0.01	*0.14* ± *0.01*	0.97 ± 0.01	0.89 ± 0.02	0.84 ± 0.03	0.94 ± 0.01
Week 12	0.15 ± 0.01	0.30 ± 0.01	0.39 ± 0.01	*0.17* ± *0.01*	0.97 ± 0.01	0.95 ± 0.01	0.99 ± 0.01
Week 16	0.14 ± 0.01	0.23 ± 0.01	0.32 ± 0.01	0.40 ± 0.01	*0.21* ± *0.02*	0.99 ± 0.00	0.99 ± 0.00
Week 20	0.15 ± 0.01	0.18 ± 0.01	0.25 ± 0.01	0.34 ± 0.01	0.42 ± 0.01	*0.21* ± *0.02*	0.97 ± 0.01
Week 24	0.14 ± 0.01	0.21 ± 0.01	0.24 ± 0.01	0.25 ± 0.01	0.27 ± 0.01	0.31 ± 0.01	*0.12* ± *0.01*

### Persistency

Table 1 shows that the production peak occurred on average during week 3 of lay. Persistency is defined as the slope from the peak to a later point in the production period. For example, the slope could be the difference in production at week 3 from the production at week 18, divided by 16, with a smaller slope representing greater persistency. Hens could be ranked by total 24-week production, within that by persistency, and within that by delay time from light date to the day of first egg or by using an index approach. This requires solving a three-trait selection problem on the components of egg production. Availability of individual feed intake would allow a complete picture on how to select efficient birds. Furthermore, RR models allow accurate predictions of breeding values of 24-week egg production based on production data over 16 to 20 weeks after first egg. Correlations between egg production based on 16 and 24 weeks were high and ranged from 0.94 to 96.

### Genetic correlations between weeks of lay

Estimates of the genetic correlations between weeks of lay were positive and ranged from moderate (0.67) to high (0.99) (Table 6). The genetic correlation estimates tended to decrease as the time interval between weeks of production increased. The lowest estimates (0.66) were found between weeks 1 and 18. Kranis et al. [24] compared RR models with multi-trait models over five consecutive 28-day periods of lay in turkeys and reported lower genetic correlations of the first with the third (− 0.09) and fourth (− 0.08) periods than those estimated in our study. Estimates of genetic correlations between periods in the later stages of production were stronger than those between earlier stages, with estimates ranging from 0.94 to 0.99. Estimates of genetic correlations for later stages of production were also similar to those found in turkeys and quail [28]. These results show that variance components and genetic correlations between records at different weeks were less than 1 and changed over time. In these situations, RR models allow more precise modelling of the data and are, therefore, recommended for the genetic evaluation of egg production traits in turkeys.

### Genomic prediction

**Fig. 1:**
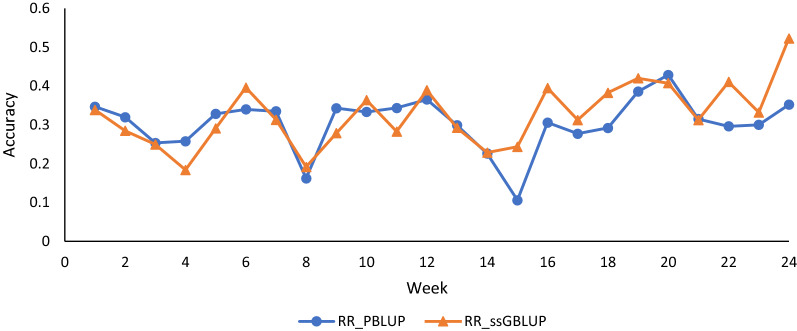
Correlation-based accuracies of estimated breeding values using single-step random regression (RR-ssGBLUP) and using pedigree-based random regression (RR-PBLUP) for egg production from weeks 1 to 24 of production

Here, comparisons of EBV and GEBV were conducted based on validation correlations and based on the variance of Mendelian sampling predictions. The validation correlations are shown in Fig. 1 for each week. The prediction accuracies ranged from 0.11 to 0.44 for RR-PBLUP and from 0.22 to 0.57 for RR-ssGBLUP. A similar advantage of ssGBLUP over the pedigree-based EBV in terms of accuracy was reported in commercial layers for the later periods of production [29].

The highest accuracies recorded in our study were at weeks 20 and 24 for RR-PBLUP and RR-ssGBLUP, respectively. The accuracies of RR-ssGBLUP were lower but not statistically different from those for RR-PBLUP for some weeks. In a previous study conducted on layer hens, genomic information did not improve the accuracy of prediction for longitudinal egg numbers in the early stages of production [29]. These results may be due to differences in the genetic architecture of egg production traits between species or breeds, differences in linkage disequilibrium (LD) between markers and QTL, and variation of the genetic expression of egg production at different ages. Further investigation into the dynamics of the prediction accuracy in different periods of egg production is ongoing.

**Fig. 2:**
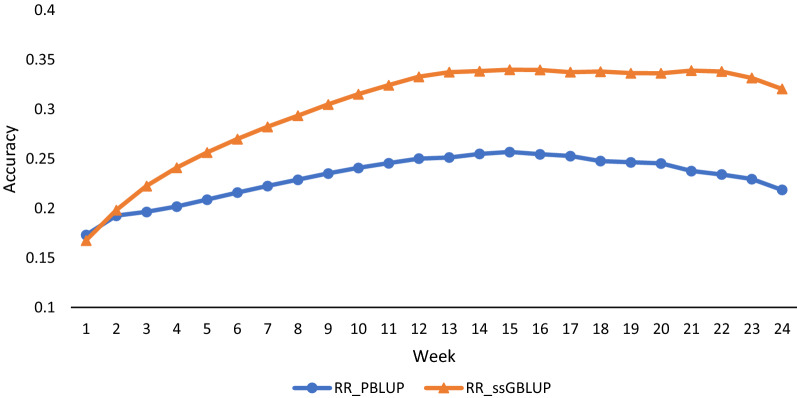
Variance of predictions of Mendelian sampling effects as a fraction of one half of the additive genetic variance based on single-step genomic random regression (RR-ssGBLUP) and pedigree-based random regression (RR-PBLUP) for egg production from weeks 1 to 24 of production

Mendelian sampling variance ratios are shown in Fig. 2 for each week. The ratios were higher for RR-ssGBLUP (ranging from 0.17 to 0.34) than for RR-PBLUP (ranging from 0.17 to 0.26), which indicates that including marker information indeed improved the estimates of Mendelian sampling effects. The gain was consistent over all weeks, as found by Buch et al. [30], who reported that prediction accuracy increased significantly for traits with a low heritability when genomic information was included. However, the accuracy of genomic prediction depends on many factors, including population size, heritability of the trait, number of markers, LD, and the number of QTL influencing the trait [29, 30]. Similar to our study, Schaeffer et al. [31] used the variance of Mendelian sampling effect predictions to compare the accuracy of genomic over pedigree analyses in Atlantic salmon and found that the genomic method gave substantially higher ratios than the pedigree method.

Both the correlation-based statistic and Mendelian sampling statistic indicate that inclusion of genomic information can improve the accuracy of predictions of breeding values and that the increase in accuracy can occur over the entire trajectory of egg production. The correlation-based statistic required the use of validation phenotypes, which include genetic, permanent environmental effect, and residual effects for a generally small subset of the data. Thus, the correlation-based statistic shows more variability over the 24 week trajectory because each time point involves a different set of phenotypic records. The Mendelian sampling statistic makes use of the same information at each time point, and thus, appears to be smooth over the production trajectory.

## Conclusions

To provide knowledge on egg production traits and maximize the accuracy of EBV for egg production in a turkey population, a single step RR model was compared with a pedigree-based RR model, and variance components were estimated over time. Although the increase in accuracy over RR-PBLUP was not uniform across time, the predictions based on RR-ssGBLUP generally increased the accuracy of prediction, especially in the later weeks of lay. Random regression models require that egg production is monitored from the day of first egg, which may need some adjustments in the procedures applied in a commercial enterprise, but in return they provide a clearer picture of the shape of the egg production curves and allow quantification of persistency of production. The economic benefits versus the cost of obtaining those benefits require further consideration.

## Data Availability

The data that support the findings of this study are available from Hybrid Turkeys upon reasonable request, but restrictions apply to their availability, since they were used under a license of a material transfer agreement for the current study, and thus are not publicly available.
